# Influence of fecal collection conditions and 16S rRNA gene sequencing at two centers on human gut microbiota analysis

**DOI:** 10.1038/s41598-018-22491-7

**Published:** 2018-03-12

**Authors:** Jocelyn Sietsma Penington, Megan A. S. Penno, Katrina M. Ngui, Nadim J. Ajami, Alexandra J. Roth-Schulze, Stephen A. Wilcox, Esther Bandala-Sanchez, John M. Wentworth, Simon C. Barry, Cheryl Y. Brown, Jennifer J. Couper, Joseph F. Petrosino, Anthony T. Papenfuss, Leonard C. Harrison, ENDIA Study Group*, Peter G. Colman, Andrew Cotterill, Maria E. Craig, Elizabeth A. Davis, Mark Harris, Aveni Haynes, Lynne Giles, Grant Morahan, Claire Morbey, William D. Rawlinson, Richard O. Sinnott, Georgia Soldatos, Rebecca L. Thomson, Peter J. Vuillermin

**Affiliations:** 1 The Walter and Eliza Hall Institute of Medical Research, Parkville, Victoria 3052 Australia; 2 Department of Medical Biology, University of Melbourne, Victoria, 3010 Australia; 3 Robinson Research Institute, University of Adelaide, Adelaide, 5006 South Australia Australia; 4 Alkek Center for Metagenomics and Microbiome Research, Department of Molecular Virology and Microbiology, Baylor College of Medicine, Houston, TX 77030 USA; 5 Department of Diabetes and Endocrinology, Royal Melbourne Hospital, Parkville, Victoria Australia; 6 Endocrinology Department, Lady Cilento Children’s Hospital, Brisbane, Queensland Australia; 7 Institute of Endocrinology and Diabetes, The Children’s Hospital at Westmead, Westmead, New South Wales Australia; 8 Serology and Virology Division, NSW Health Pathology, Prince of Wales Hospital, Randwick, New South Wales Australia; 9 University of NSW, Sydney, Australia; 10 Department of Endocrinology & Diabetes, Princess Margaret Hospital for Children, Perth, Western Australia Australia; 11 Telethon Kids Institute, Subiaco, Western Australia Australia; 12 School of Public Health, University of Adelaide, Adelaide, South Australia Australia; 13 Centre for Diabetes Research, Harry Perkins Institute of Medical Research, Nedlands, Western Australia Australia; 14 Hunter Diabetes Centre, Mereweather, New South Wales Australia; 15 Melbourne eResearch Group, University of Melbourne, Parkville, Victoria Australia; 16 Diabetes and Vascular Medicine Unit, Monash Health, Clayton, Victoria Australia; 17 Adelaide Medical School, University of Adelaide, Adelaide, South Australia Australia; 18 Child Health Research Unit, Barwon Health, Geelong, Victoria Australia

## Abstract

To optimise fecal sampling for reproducible analysis of the gut microbiome, we compared different methods of sample collection and sequencing of 16S rRNA genes at two centers. Samples collected from six individuals on three consecutive days were placed in commercial collection tubes (OMNIgeneGut OMR-200) or in sterile screw-top tubes in a home fridge or home freezer for 6–24 h, before transfer and storage at −80 °C. Replicate samples were shipped to centers in Australia and the USA for DNA extraction and sequencing by their respective PCR protocols, and analysed with the same bioinformatic pipeline. Variation in gut microbiome was dominated by differences between individuals. Minor differences in the abundance of taxa were found between collection-processing methods and day of collection, and between the two centers. We conclude that collection with storage and transport at 4 °C within 24 h is adequate for 16S rRNA analysis of the gut microbiome. Other factors including differences in PCR and sequencing methods account for relatively minor variation compared to differences between individuals.

## Introduction

The fecal or ‘gut’ microbiome is shaped strongly by diet but also by the host genotype, age, hygiene and antibiotic exposure, and is altered in many pathophysiological states^1,2^. The composition of gut microbiota differs greatly between individuals^3^ and therefore maximizing the detection of biological and disease-related changes requires minimization of variation due to methods of sample collection and analysis.

Previous studies have shown that fecal microbial composition overall was not altered when DNA was extracted from a fresh fecal sample compared to a sample that had been immediately frozen and stored at −80 °C for up to 6 months^4,5^. Storage at different temperatures for varying times has been compared with immediate freezing and storage at −80 °C. One study^5^ reported a decrease in *Bacteroidetes* and an increase in *Firmicutes* phyla after 30 minutes at room temperature, but the majority^4,6–11^ have found that storage at room temperature for at least a day, or at 4, −20 or −80 °C for up to 14 days, had little effect on the relative abundance of taxa. Moreover, microbial composition was not significantly altered in DNA extracted from fecal occult blood test cards that had been at room temperature for three days^12^. Recent studies have also evaluated the OMNIgene®•GUT (OMR-200) collection and liquid storage tube, which is reported to stabilize DNA at room temperature for 14 days^13^. Samples immediately collected into these tubes and stored for three days at room temperature showed little difference in microbial composition by 16S rRNA gene sequencing compared with samples immediately frozen at −80 °C^7^. A similar result was obtained when samples in OMR-200tubes stored for 1–28 days at room temperature were compared with samples from which DNA was freshly extracted^14,15^. However, the relative abundance of *Bacteroides* increased after seven days and in infants (who had lower microbial diversity than adults) significant differences from fresh samples were observed after 14 days storage.

As microbiome studies expand into larger populations at multiple sites, stringent quality control remains critical. With this in mind and to optimise analysis of the gut microbiome in a multi-site, longitudinal pregnancy-birth cohort study^16^, we evaluated the impact of different collection-processing methods on three sequential daily fecal samples from six individuals for 16S rRNA gene sequencing in two centers.

## Methods

**Figure 1:**
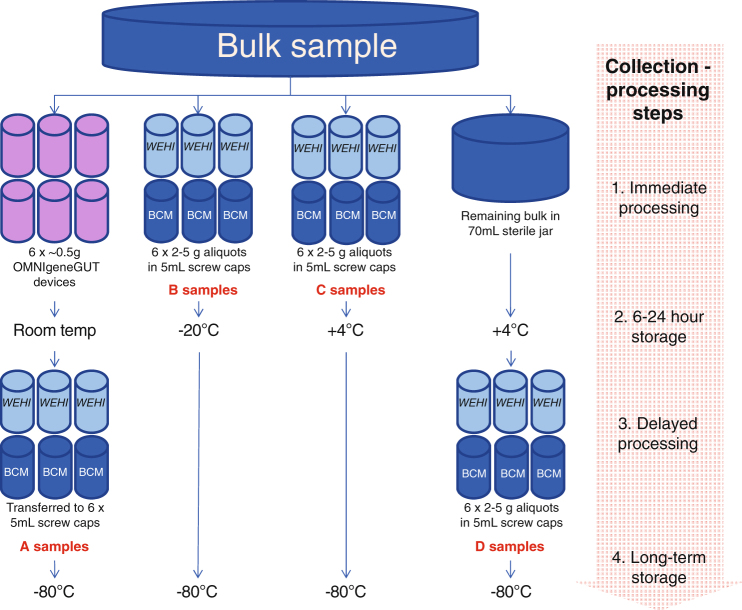
Schematic of the four collection methods for a home-collected fecal sample. Six individuals collected samples over three days. Each sequencing center received three aliquots per person-day-method combination (216 in total).

Six healthy adult volunteers, three males and three females aged 35–70, provided fecal samples on three successive days. Multiple aliquots were taken from each bulk sample and those for bacterial microbiome analysis were stored by one of four methods A-D (Fig. 1).

### Collection-processing methods

Method A: individuals placed aliquots of feces into 6× OMNIgene®•GUT (OMR-200)^13^ tubes as per manufacturer’s protocol. These were stored at room temperature for 6–24 h before delivery to a laboratory for transfer to sterile 5 mL screw cap tubes and storage at −80 °C. Method B: individuals placed aliquots of feces into 6× sterile 5 ml screw cap tubes, which were stored in the home freezer for 6–24 h before delivery to the laboratory in an insulated container for storage at −80 °C. Method C: individuals placed aliquots of feces into 6× sterile 5 m screw cap tubes, which were stored in the home refrigerator for 6–24 h before delivery to the laboratory in an insulated container for storage at −80 °C. Method D: individuals placed a bulk fecal sample into a sterile 70 ml collection jar, which was stored in home refrigerator for 6–24 h before delivery to the laboratory in an insulated container, transfer of aliquots into 6× sterile 5 m screw cap tubes and storage at −80 °C.

In the laboratory, samples were handled under sterile conditions in a Biosafety Level 2 cabinet. This collection-processing procedure yielded a total of 432 samples (24 from each of 6 individuals per day for 3 days) (Fig. 1). After 2–4 weeks at −80 °C, three sample aliquots per person-day-method (n = 216) were transported on dry ice to sequencing centers in Australia (Walter and Eliza Hall Institute of Medical Research, Melbourne, Victoria; WEHI), and the USA (Baylor College of Medicine, Alkek Center for Metagenomics and Microbiome Research, Houston, Texas; BCM), for 16S rRNA gene amplicon sequencing. At both WEHI and BCM samples were stored at −80 °C for 4 weeks before sequencing.

### DNA sequencing methods

Samples were thawed on ice and DNA extracted at both WEHI and BCM with the MoBio PowerSoil kit (MoBio Laboratories, Carlsbad, CA), as in the Human Microbiome Project^17^. At both WEHI and BCM, the V4 hypervariable region of the bacterial 16S rRNA marker gene (16Sv4) was PCR-amplified with primers 515F-OH1 and 806R-OH2. Analogues of these are described, respectively, by the common name U515F, new name S-*-Univ-0515-a-S-19, and the common name 806R, new name S-D-Bact-0787-b-A-20 in^18^.

At WEHI, these primers (GTGACCTATGAACTCAGGAGTCGGACTACNVGGGTWTCTAAT) and (CTGAGACTTGCACATCGCAGCGTGYCAGCMGCCGCGGTAA) included unique sequences (underlined) to provide a target for the subsequent introduction of Illumina sequencing adaptors and dual index barcodes to the amplicon target for paired-end sequencing on the Illumina MiSeq instrument^19^. Primary 16S rRNA gene PCR amplification was performed in duplicate with the following conditions: 94 °C for 3 minutes followed by 20 cycles at 94 °C for 45 seconds each, 55 °C for 1 minute, and 72 °C for 1 minute 30 seconds and a final extension step at 72 °C for 10 minutes. Successful amplification was determined by agarose gel electrophoresis. Amplicons from the primary amplification were diluted 1/10 and used as template for the secondary amplification. In the secondary amplification, the overhang sequences were used to introduce Illumina sequencing adaptors and dual index barcodes to the amplicon target. Individual amplicons were identified using 8 base index sequences from the Illumina Nextera design. Sixteen forward index primers and 24 reverse index primers were designed for a 96-well plate format with the potential to generate a maximum of 384 dual index amplicons. PCR conditions were as for the primary amplification, except for an increase to 25 cycles. Reactions were performed in triplicate in separate plates, with extra wells added, up to a maximum of 9 wells, if PCR product appeared low. Amplicon size distribution was determined by agarose gel electrophoresis. One sample failed to yield sufficient product in any well. Reactions from the three replicate plates were pooled and the PCR amplicons purified with 1.0× NGS Beads (Macherey-Nagel). Each dual indexed library plate pool was quantified with the Agilent Tapestation and the Qubit™ DNR BR assay kit for Qubit 3.0® Fluorometer (Life Technologies). The indexed pool was diluted to 12pM and sequenced with the paired end 600-cycle (2 × 311) kit on a MiSeq instrument. After quality filtering, 702 technical replicate samples comprising 215 biological samples from the 6 individuals were obtained.

At BCM, the 16S V4 sequencing methods were adapted from the NIH-Human Microbiome Project^17^ and the Earth Microbiome Project^20,21^. The primers used for amplification contained adapters for MiSeq sequencing and single-end barcodes allowing pooling and direct sequencing of PCR products^21^. PCR-amplified amplicons were normalized by concentration before pooling and sequences were generated in one lane of a MiSeq instrument using the v2 kit (2 × 250 bp paired-end protocol). The 216 samples were analysed in a single pool.

### Bioinformatic analysis

Sequences were clustered into operational taxonomic units (OTUs) with 97% similarity using QIIME (Version 1.8.1)^22–25^, and taxonomically classified by aligning the representative sequences to the Greengenes 13_08 database^26^. Paired-end sequences were assembled with PEAR^27^ with parameters -v 100 -m 600 -n 80, where -v is minimum overlap, -m is maximum assembled length and -n is minimum assembled length. WEHI index sequences were extracted with QIIME script extract_barcodes.py, and bases up to and including the 533F to 805R V4 region amplicon primers were trimmed. BCM sequences were supplied as trimmed sequences with a separate barcode index file.

QIIME’s split_libraries_fastq.py script was used for quality filtering, with Phred quality scores required to be above 29, and 90% of a read’s length required to have consecutive, high-quality base calls. In order to minimize differences between WEHI and BCM sequences, WEHI sequences were further filtered by aligning to the SILVA 123 16S rRNA gene database using MOTHUR v1.38.1^28,29^ with start = 8390 and end = 17068, and by removing sequences that were not in this position.

QIIME’s open-reference OTU-picking was used with the Uclust algorithm^22,23^ to form clusters at 97% similarity. The representative sequences from each OTU were aligned with gaps to a reference set using QIIME’s implementation of PyNAST, then filtered for chimeric sequences using UChime with default settings^30^. After making a filtered OTU table with minimum count 3, and assigning taxonomy with Greengenes_13_08^26^, the data were analyzed in R.

WEHI data were derived from 759 (PCR-well) samples. Those with sequence counts <1000 or with descriptions of PCR products as ‘None’ or ‘Very faint’ were discarded, which included all samples for individual 55, method B, day 3, aliquot 3. OTU counts for the remaining technical replicates were summed to give 215 biological-replicate samples.

Alpha and beta diversity and differential abundance analyses were performed in R using the Phyloseq package^31^ and DESeq2^32^. Most analyses used 215 or 216 samples with minimum OTU size of 20; alpha diversities were calculated with the minimum-count = 3 OTUs with technical and biological replicates combined, giving 72 samples.

### Data availability

The sequences analysed during this study are available in the ENA Short Read Archive, with accession PRJNA393083 (SRP116702), https://www.ncbi.nlm.nih.gov/bioproject/?term=PRJNA393083. The OTU tables generated are available through Figshare with accession 5401594, https://figshare.com/articles/ENDIA_microbiome_QC_OTUs_and_metadata/5401594.

## Results

For the WEHI data set, library sizes after quality filtering, clustering, and combining PCR replicates ranged from 30,000 to 250,000 sequences per sample, with a median of 67,000 (Figure S1A); sequences clustered into 12,652 OTUs of minimum size 20. For the BCM data set, library sizes ranged from 5,000 to 56,000, with a median of 27,000 (Figure S1B); sequences clustered into 3,675 OTUs of minimum size 20. The final number of sequences reflects differing sequencing and filtering protocols, including the use of multiple PCR replicates at WEHI.

### Taxonomic overview

**Figure 2:**
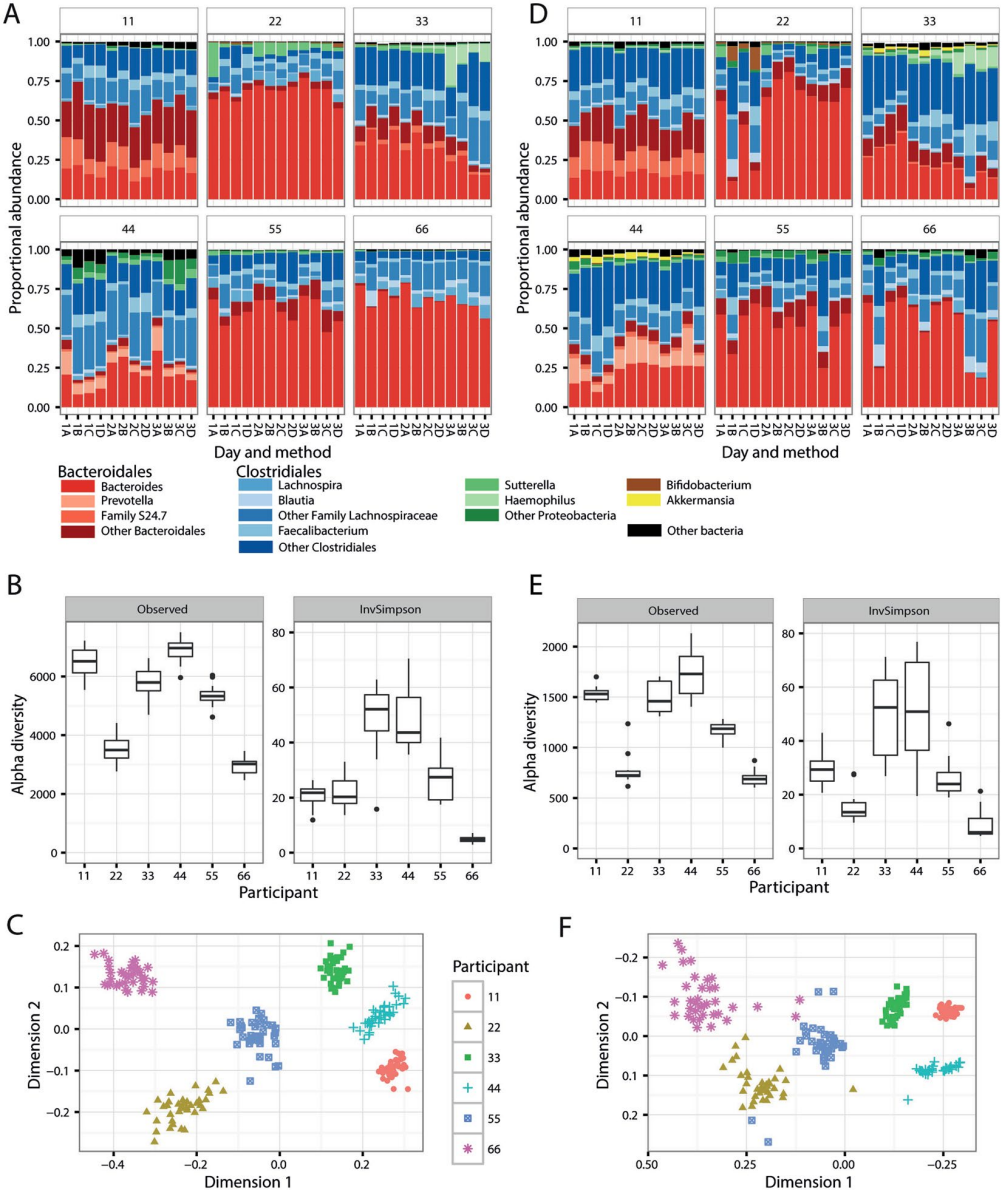
Overview of the fecal bacterial microbiome from sequencing at WEHI **(A,B,C)** or BCM **(D,E,F)**. (**A,D**) Dominant bacterial genera in fecal samples, or higher taxa where genus was not available. Bars are colour-coded by phyla: red *Bacteroidetes*, blue *Firmicutes*, green *Proteobacteria*, brown *Actinobacteria*, yellow *Verrucomicrobia*. (**B,E**) Alpha diversity within samples. Two measures are shown: observed number of OTUs per sample, an estimate of richness, and Inverse-Simpson index indicating the evenness of the sample. Samples were sub-sampled to the smallest sample size, and values are the mean of 10 random sub-samples. Boxes show the inter-quartile range for the four methods on three days. (**C,F**) Beta diversity. NMDS ordination of the UniFrac distance between samples, a representation of phylogenetic similarity.

Samples were dominated at the phylum level by *Bacteroidetes* and *Firmicutes*, as expected. The mean summed proportion of these two phyla was 94%, varying from 71.9% to 99.7% between individual samples. Likewise, a single order from each of these two phyla, *Bacteroidales* and *Clostridiales*, was dominant, with three orders from the phylum *Proteobacteria* contributing another 1–2% overall (Fig. 2A,D; see also Figure S2).

Alpha (α) diversity is used to characterise the richness of the microbiome and its evenness (heterogeneity) or distribution of proportions. Samples showed a considerable spread of α diversity (Fig. 2B,E). Samples from individual 66 had the lowest observed richness (number of OTUs per sample) and the lowest Inverse-Simpson diversity index, the latter indicating dominance by a smaller number of OTUs. This is reflected in the genera plots (Fig. 2A,D). In contrast, samples from individual 11 had a high observed richness but a comparatively low Inverse-Simpson index, consistent with the presence of a few high-abundance and multiple low-abundance genera.

Analysis of β diversity by non-metric multidimensional scaling (NMDS) ordination of the UniFrac distance showed that samples cluster strongly by individual, with marked separation between individuals (Fig. 2C,F).

### Differences between WEHI and BCM data sets

For WEHI and BCM data sets the most abundant phyla were similar, but the proportions of less abundant phyla and higher taxonomic resolution differed. For example, the mean proportion of genus *Akkermansia* in the order *Verrucomicrobiales* was greater in BCM (0.7%) than WEHI (0.02%). The proportion of *Bacteroides* was lower in some individual samples for BCM than WEHI (Fig. 2B; also S2).

The BCM data set yielded fewer OTUs and therefore had lower values for observed richness (Fig. 2B,E). The number of OTUs observed per sample was dependent on sampling depth (Figure S3); values shown are based on the smallest sample sizes for each of the two data sets. Richness was similar between the WEHI and BCM data sets, with samples from individual 66 showing the lowest alpha diversity and those from individual 44 the highest. For the Inverse-Simpson diversity index, which is not dependent on library size at this depth of sequencing, the BCM data set had a greater range of values, and a greater range for samples from some individuals. Both data sets had similar patterns of beta diversity between individuals (Fig. 2C,F), although the BCM data set had several outliers.

**Figure 3:**
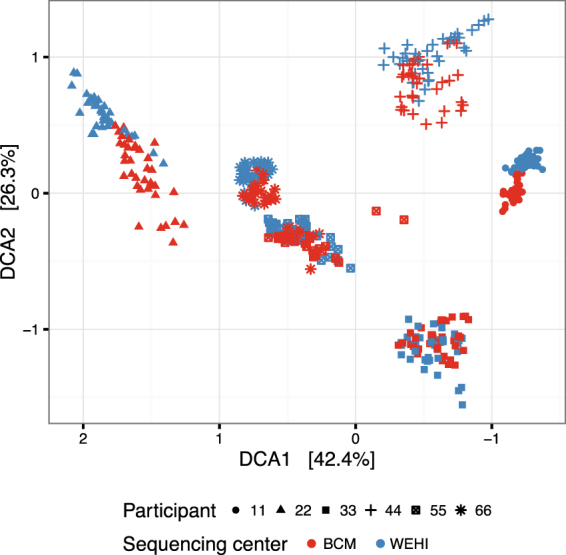
Beta diversity between samples from two sequencing centers. Ordination plot of Bray-Curtis distances between samples, using Detrended Correspondence Analysis. Points represent samples from BCM and a single technical replicate from WEHI.

Initial analysis was performed separately on the WEHI and BCM datasets. For better comparison of the taxonomies, the bioinformatic pipeline was re-applied to a data set comprising the BCM sequences and one of the three WEHI technical replicates (Fig. 3). The ordination plot shows ‘batch’ effects between the two sequencing centers and greater between-sample differences in the BCM data set.

DESeq2 was used to make generalized linear models for the counts at phylum, order and OTU levels (Table 1). The model included individual ID, day and collection-processing method as factors. At the phylum level, the largest change was in the *Verrucomicrobia*. At the OTU level, 3% of OTUs were significantly different (Figure S4, Additional data S1). Most of the differentially abundant OTUs belonged to the orders *Clostridiales* (63%) and *Bacteroidales* (31%). The direction of change in OTUs was not consistent, and there were no significant differences in counts for *Clostridiales* and *Bacteroidales* between WEHI and BCM data sets.

**Table 1:** Analysis of combined data from BCM and WEHI.

Phylum	Base mean	Log_2_ (fold change)	Adjusted p-value	Max proportion WEHI	Max proportion BCM
*Verrucomicrobia*	39.2	−2.9	2.6 × 10^−39^	1.1%	5.1%
*Actinobacteria*	68.7	−1.5	3.5 × 10^−06^	3.6%	21.3%
*Lentisphaerae*	9.6	1.6	1.5 × 10^−02^	1.3%	0.3%
**Order**					
*Verrucomicrobiales* (V)	38.7	−3.2	4.8 × 10^−45^	0.3%	5.1%
*Enterobacteriales* (P)	146.4	−2.0	4.3 × 10^−10^	8.4%	9.7%
*Actinomycetales* (A)	0.8	−2.0	2.4 × 10^−07^	0.03%	0.1%
*Victivallales* (L)	10.4	2.0	9.6 × 10^−06^	1.3%	0.3%
*Bifidobacteriales* (A)	44.1	−1.4	6.8 × 10^−03^	3.5%	20.9%

Log_2_ (WEHI/BCM) is based on a fitted mean of the counts in samples as calculated by the DESeq2 package^32^. The maximum proportions are included for context. The adjusted p-value is from a Wald test with the default BH adjustment. For each order the phylum is indicated in brackets: A *Actinobacteria*, L *Lentisphaerae*, P *Proteobacteria*, V *Verrucomicrobia*.

### Effect of collection-processing method on taxonomic analysis

**Figure 4:**
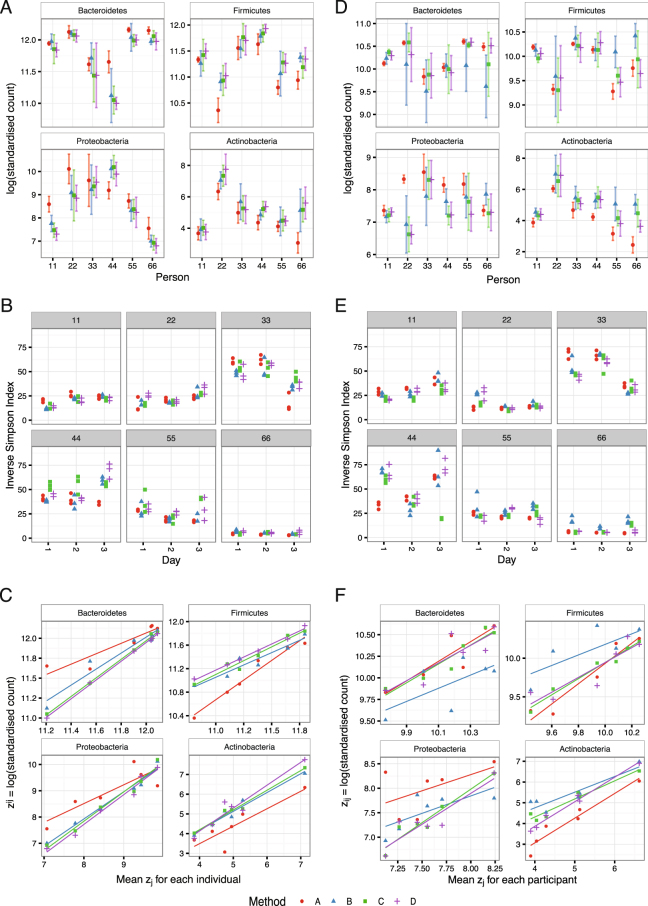
Effect of collection-processing method from sequencing at WEHI (**A**,**B**,**C**) or BCM (**D**,**E**,**F**). (**A,D**) Log of standardised counts (scaled by library size) of the four most abundant phyla. Points show mean and bars standard deviation (sd) for each individual and collection-processing method. Method A has the smallest average sd for *Bacteroidetes* and *Actinobacteria*. (**B,E**) The Inverse-Simpson α diversity index for each sample (compare with Fig. 2). (**C,F**) Mean log (standardised count) plotted against the mean over the collection-processing methods, and a linear regression applied. Method A has the greatest average deviation from the linear model for the WEHI data set.

Testing of the WEHI data set for differential abundances between collection-processing methods, using DESeq2 with a design controlling for the effect of person and day, revealed no significant differences in counts by phylum, order or family (Table 2, Fig. 4A). Five OTUs (0.04% of OTUs comprising 0.2% of sequences) were different under collection-processing Method A. With the BCM data set, collection-processing Methods A and B were taxonomically different, with a decrease in *Actinobacteria* in Method A (Fig. 4D) and an increase in *Lentisphaerae*, although counts were very low (p < 0.001, Additional Table S1). *Lentisphaerae* were also increased in Method A compared with Methods C and D (p < 0.05).

**Table 2:** Differences by collection-processing method in phyla and OTU counts (WEHI and BCM data sets). No collection-processing method had significantly different phyla in the WEHI data set.

Method comparison	# OTUs different (WEHI)	# phyla different (BCM)	# OTUs different (BCM)
A-D	1	1	26
B-D	0	0	0
C-D	0	0	0
B-A	0	2	51
C-A	5	1	25
C-B	0	0	0

Wald test for absolute value of log_2_ (fold change) > 1 with Benjamini-Hochberg adjusted p-value < 0.05.

Diversity varied within a sample depending on collection-processing method (Fig. 4B,E) but the effect was small and inconsistent. After fitting a linear model with inputs for method and individual, 20–30% of variation was unaccounted for, while collection-processing method accounted for only 2%. Overall, alpha diversity was slightly lower with Method A in the WEHI data set, and higher with Method B in the BCM dataset. (Table 3).

**Table 3:** Change in α diversity (Inverse-Simpson index) due to collection-processing method.

Method comparison	Difference in Inv-Simpson Index (WEHI)	Family-wise adjusted p value (WEHI)	Difference in Inv-Simpson Index (BCM)	Family-wise adjusted p value (BCM)
A-D	−3.8	0.07	−0.7	0.99
B-D	−2.9	0.24	3.5	0.29
C-D	0.6	0.97	−3.9	0.21
B-A	0.9	0.95	4.2	0.15
C-A	4.4	0.02	−3.2	0.38
C-B	3.6	0.10	−7.4	0.001

Different methods of collection-processing might also increase the variance between samples, reducing the reproducibility of a result. Two approaches were used to test for this. Greater variance between samples is equivalent to greater distance between samples by some measure. The Bray-Curtis dissimilarity between OTU counts was calculated for pairs of samples from each individual and method, and a Tukey Honest Significant Difference test applied to a linear model of the dissimilarity. There was no evidence that the dissimilarity between samples was different for collection-processing methods (smallest p = 0.1) in the WEHI data set. There were significant differences in Bray-Curtis distances between samples in the BCM data set (p < 0.001), with collection-processing Method A associated with smaller differences between samples from the same individual than Methods B, C and D (Additional Table S2).

In addition, we looked for differences in the variance of the four most abundant phyla. The log transformed standardised counts for *Bacteroidetes*, *Firmicutes*, *Proteobacteria* and *Actinobacteria* per sample were compared with the mean across collection-processing methods for each individual (Fig. 4C,F). In the WEHI data set Methods B, C and D gave similar results, while method A had lower variance within samples from the same individual but greater deviation from the mean compared with the other methods.

### Effect of collection-processing method on library size

Collection-processing methods were compared after quality filtering, barcode extraction and clustering. In the WEHI data set, the number of DNA sequences extracted per sample was not different by collection-processing method; in the BCM data set, collection-processing Method D resulted in fewer sequences than other collection-processing methods, but the difference was small compared with total variation (Figure S5). Batch effects (sequencing run) were more significant (p < 10^−5^) than collection-processing method, but batch and method together contributed less than 5% of the variation in library size.

## Discussion

This was a quality assessment study prior to commencing a multi-site, longitudinal pregnancy-birth cohort study. Our main focus was the comparison of fecal sample collection and storage methods, with samples sequenced at two centers. Not unexpectedly, given between-center differences in DNA extraction and sequencing methodologies, some differences in WEHI and BCM data sets were observed. For example, the proportions of phyla *Verrucomicrobia* and *Actinobacteria*, and order *Enterobacteriales* were increased in the BCM compared to WEHI data set. Although samples were transported and arrived on dry ice at both centers, covert effects of shipping and handling can’t be excluded.

Different collection-processing methods for fecal samples were associated with only minor differences in fecal microbiota derived by 16S rRNA gene sequencing. In the BCM data set Method A using the OMNIgeneGut OMR-200 device exhibited differences in phyla Actinobacteria and Lentisphaerae proportions compared with the methods that involved either immediate freezing or refrigeration. In the WEHI data set, no method was associated with significantly more distance between samples at the OTU level. However, in the BCM data set beta diversity was reduced between Method A samples compared to the other methods, possibly reflecting mitigation of storage and transport effects by the OMR-200 device. In WEHI data set, the log-values of proportions of the four dominant phyla had lower variance within samples from the same individual, but greater deviation from the mean, for samples collected with Method A.

In conclusion, collection-processing methods and day of collection contribute to only minor variation in fecal microbiome extracted sequences, composition and diversity, the major variation being at the level of the individual. Collection with storage and transport at 4 °C within 24 h is adequate for analysis of the fecal microbiome. These findings complement and support the results of several previous studies^4–11^. They are relevant to the quality control of gut microbiome studies, in particular to population-based multi-site studies, samples from which would ideally be analysed by the same methodology.

## Declarations

### Ethics approval and consent to participate

Volunteers gave informed consent for self-collection of non-identifiable stool samples and basic, demographic information. In accordance with the Australian NHMRC National Statement on Ethical Conduct in Human Research, the research was considered as ‘negligible risk’ with no foreseeable risk of harm or discomfort to the volunteers and therefore deemed exempt from ethical review. The methods for sample collection have been approved by the Women’s and Children’s Health Network Human Research Ethics Committee under project numbers HREC/16/WCHN/66 and HREC/13/WCHN/29.

### Availability of data and materials

Additional Figures S1–S6 and Additional Tables S1 and S2 attached as Additional Figures and Tables. Additional data attached as xlsx file.

R code is available as joint_plots_tables.Rmd from github.com/PapenfussLab/endiaQC/.

## Electronic supplementary material

Additional Figures and Tables

Additional data
