# Supplementary material for: Influence of fecal collection conditions and 16S rRNA gene sequencing at two centers on human gut microbiota analysis
Source: Sci Rep. 2018 Mar 12;8:4386. doi: 10.1038/s41598-018-22491-7 (PMC5847573; doi:10.1038/s41598-018-22491-7)
Supplement: Supplementary file 1 — Additional Figures and Tables [file 41598_2018_22491_MOESM1_ESM.pdf]

Influence of fecal collection conditions and 16S rRNA gene sequencing at two centers on human gut microbiota analysis

Jocelyn Sietsma Penington[1]^ (penington.j@wehi.edu.au), Megan A S Penno[3]^, Katrina M Ngui[1]^, Nadim J Ajami[4]^, Alexandra J Roth-Schulze[1,2], Stephen A Wilcox[1], Esther Bandala-Sanchez[1], John M Wentworth[1], Simon C Barry[3], Cheryl Y Brown[3], Jennifer J Couper[3], Joseph F Petrosino[4], Anthony T Papenfuss[1,2+], Leonard C Harrison[1+] and the ENDIA Study Group (endia@adelaide.edu.au).

Legends to additional figures

Figure S1

Number of sequences in combined samples. Colours indicate the three aliquots of each sample. (**A**) Walter and Eliza Hall Institute 16S rRNA gene sequences. Horizontal black lines within aliquots indicate the PCR replicates retained after filtering, usually 3 but up to 9. Total number of sequences 15 million. (**B**) Baylor College of Medicine Human Genome Sequencing Centre (BCM) 16S rRNA gene sequences. Total number of sequences 6.0 million.

Figure S2

Proportion (log base 10) of bacterial orders in 72 samples (combining aliquots from the same stool). The X-axis is arranged by mean proportion. Order names are from Greengenes 13_08 database; square brackets indicate name proposed by the curators for an uncultured bacterium. Streptophyta is a chloroplast probably from undigested plant matter. (**A**) WEHI. (**B**) BCM.

Figure S3

Two measures of sample α diversity at multiple sub-sampling depths for two sequencing facilities. Each individual has 12 plots, corresponding to four methods by three collection days. Values are means of 8 random sub-samples, taken without replacement. Standard deviation bars are smaller than point sizes. (**A**) WEHI data set. The vertical black line shows the smallest sample. The smallest sample from BCM is near the smallest sub-sample at 31,500 sequences. (**B**) BCM data set. The vertical black line shows the smallest sample.

Figure S4

Scatter plot of OTUs showing log_2 fold changes of WEHI counts over BCM counts versus the mean normalized count. Red dots are OTUs with adjusted $p < 0.05$, triangles are points with fold changes outside the y-axis limits. The blue lines illustrate the null hypothesis that any change is less than 2-fold. Although the numbers of significant changes are evenly split between increase and decrease, WEHI counts tend to be increased in small OTUs and BCM counts in larger OTUs.

Figure S5

Library size by collection-processing method. (**A**) WEHI sequencing (**B**) BCM sequencing.

Figure S6

Overview of samples sequenced at Baylor College of Medicine and analysed with CMMR pipeline.

(**A**) Stacked bar chart of dominant bacterial genera in each sample, equivalent to Figure 4A. Bars are colour-coded by phyla using the same colours as in Figure 2. Use of the

Silva database for taxonomic assignment has introduced genus labels Prevotellaceae_UCG_001 and Lachnospiraceae_UCG_008.

(**B**) Beta diversity using UniFrac distances between samples. Axes have been reflected to give approximately the same orientation of clusters as for Figure 2F. Directions in NMDS are arbitrary, so the positioning and rotation of clusters does not indicate a real change in the UniFrac distances.

(C) Log of standardised counts (scaled by library size) of four phyla for the four methods for each individual, equivalent to Figure 3D. Points show mean and bars show standard deviation for each individual and collection-processing method (n=9).

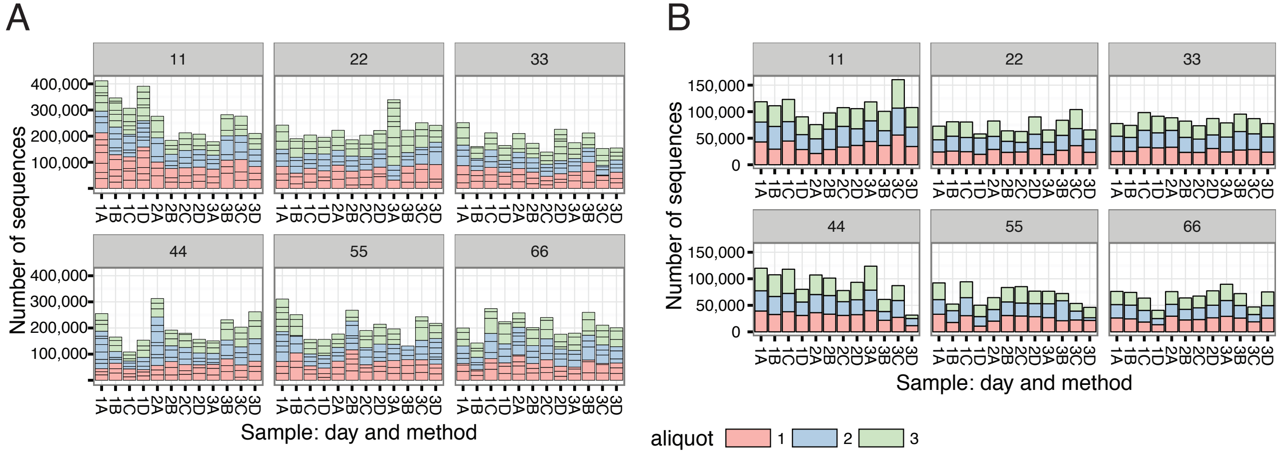
A
B
11
22
33
44
55
66
Number of sequences
Sample: day and method
aliquot
1
2
3

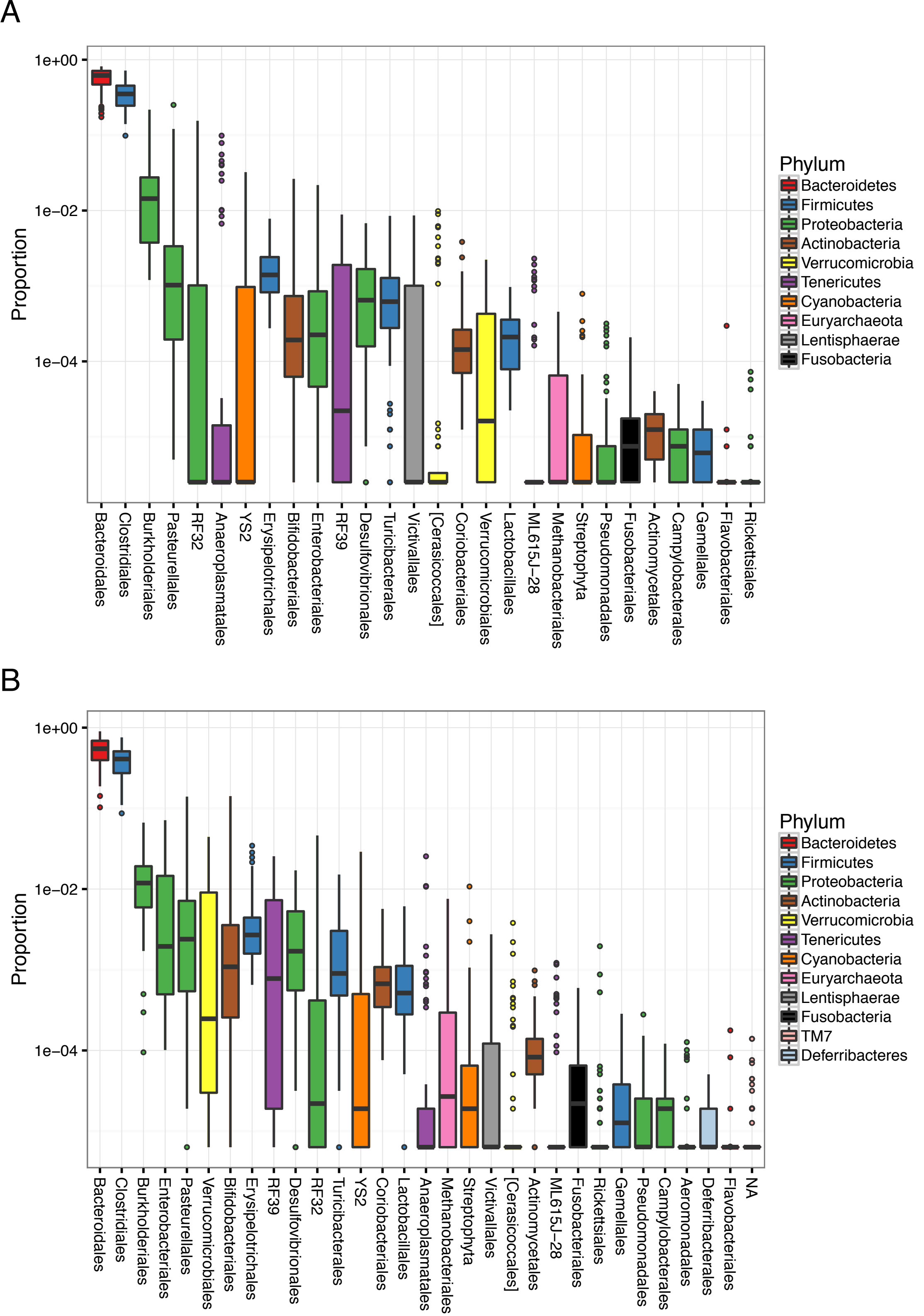

A
1e+00
1e−02
1e−04
Proportion
Phylum
Bacteroidetes
Firmicutes
Proteobacteria
Actinobacteria
Verrucomicrobia
Tenericutes
Cyanobacteria
Euryarchaeota
Lentisphaerae
Fusobacteria
Bacteroidales
Clostridiales
Burkholderiales
Pasteurellales
RF32
Anaeroplasmatales
YS2
Erysipelotrichales
Bifidobacteriales
Enterobacteriales
RF39
Desulfovibrionales
Turicibacterales
Victivallales
[Cerasicoccales]
Coriobacteriales
Verrucomicrobiales
Lactobacillales
ML615J−28
Methanobacteriales
Streptophyta
Pseudomonadales
Fusobacteriales
Actinomycetales
Campylobacterales
Gemellales
Flavobacteriales
Rickettsiales
B
1e+00
1e−02
1e−04
Proportion
Phylum
Bacteroidetes
Firmicutes
Proteobacteria
Actinobacteria
Verrucomicrobia
Tenericutes
Cyanobacteria
Euryarchaeota
Lentisphaerae
Fusobacteria
TM7
Deferribacteres
Bacteroidales
Clostridiales
Burkholderiales
Enterobacteriales
Pasteurellales
Verrucomicrobiales
Bifidobacteriales
Erysipelotrichales
RF39
Desulfovibrionales
RF32
Turicibacterales
YS2
Coriobacteriales
Lactobacillales
Anaeroplasmatales
Methanobacteriales
Streptophyta
Victivallales
[Cerasicoccales]
Actinomycetales
ML615J−28
Fusobacteriales
Rickettsiales
Gemellales
Pseudomonadales
Campylobacterales
Aeromonadales
Deferribacterales
Flavobacteriales
NA

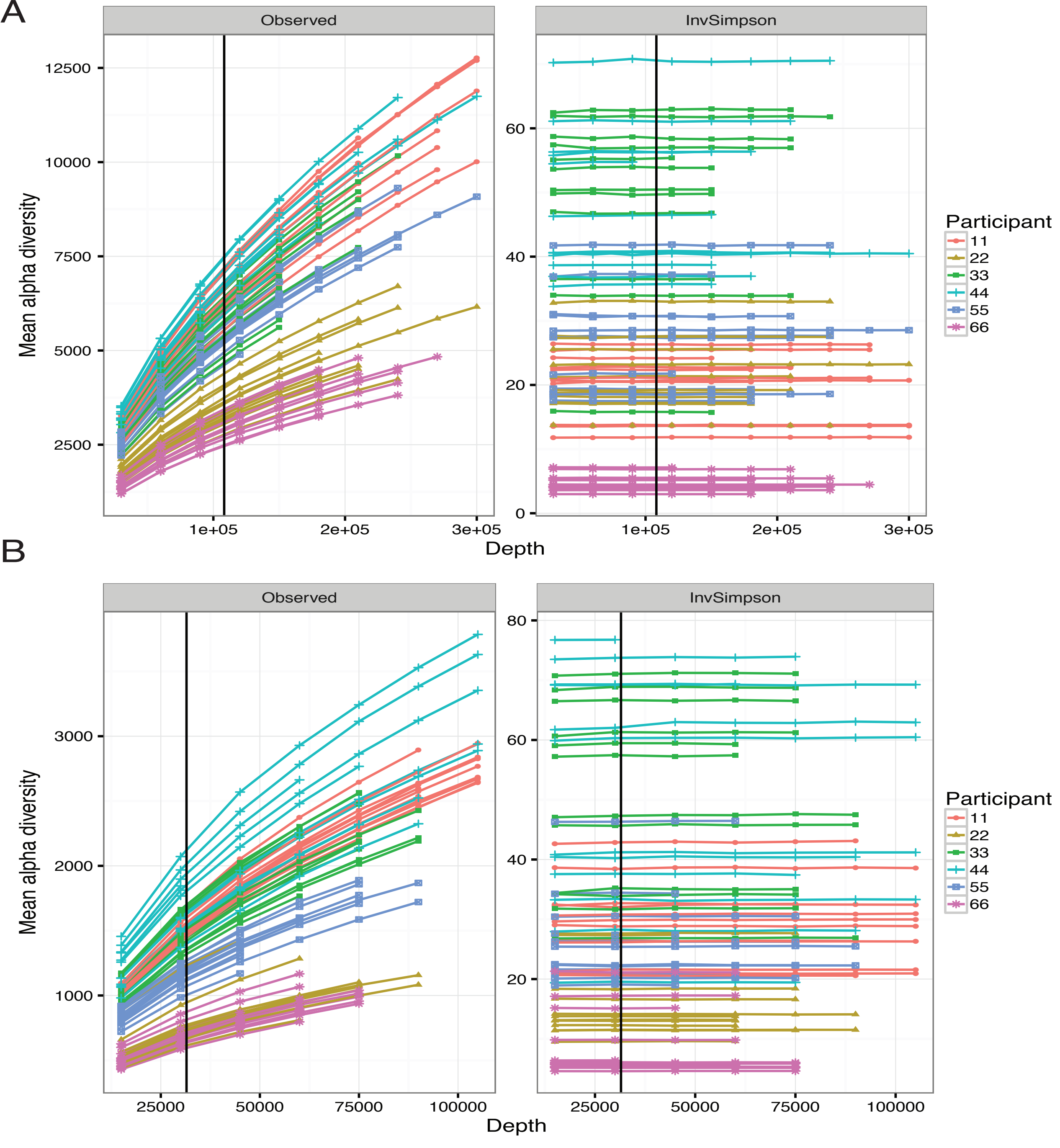
A
Observed
InvSimpson
12500
10000
7500
5000
2500
Mean alpha diversity
60
40
20
0
1e+05
2e+05
3e+05
Depth
Participant
11
22
33
44
55
66
B
Observed
InvSimpson
3000
2000
1000
Mean alpha diversity
80
60
40
20
25000
50000
75000
100000
Depth
Participant
11
22
33
44
55
66

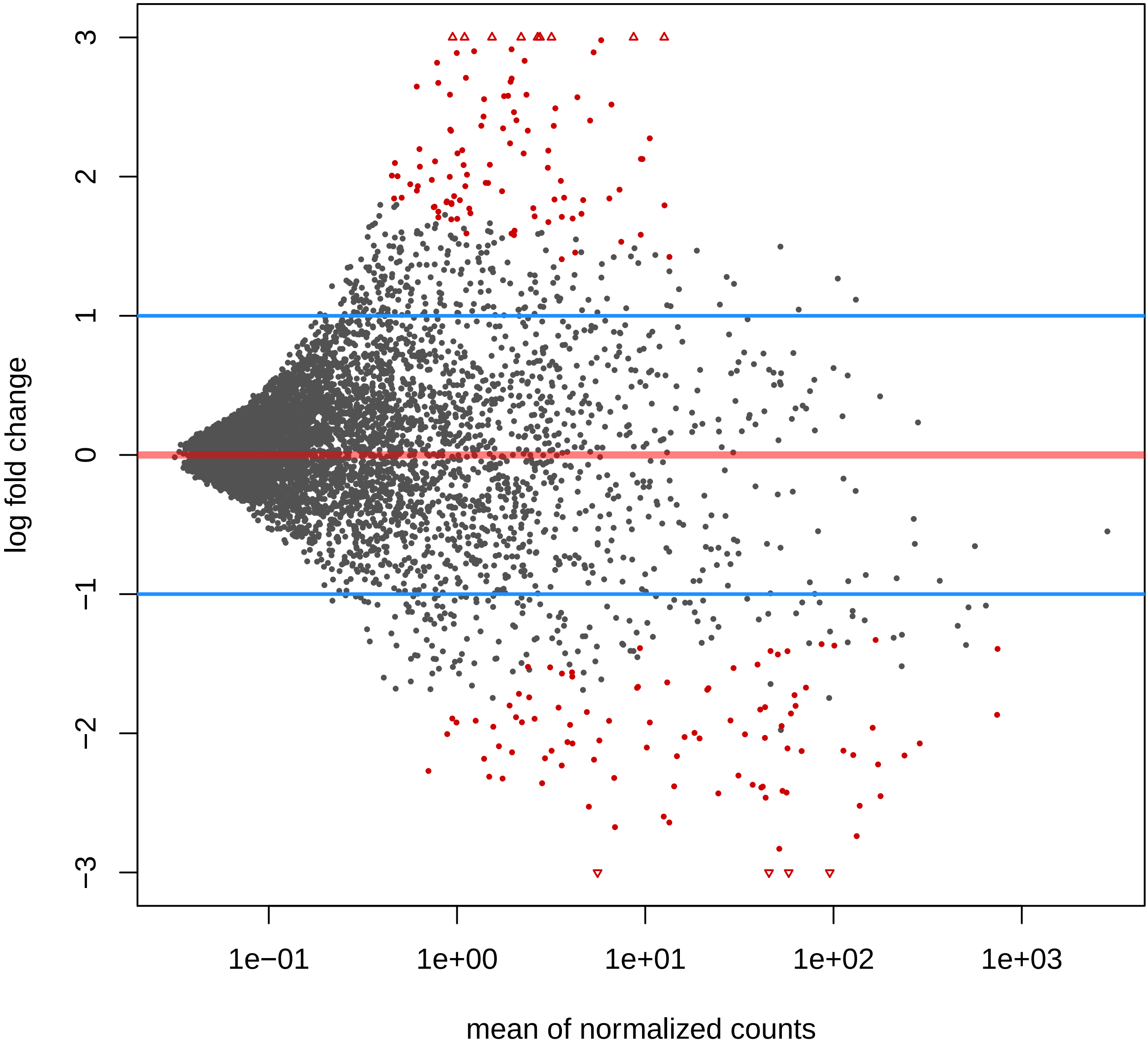

3
2
1
0
−1
−2
−3
log fold change
1e−01
1e+00
1e+01
1e+02
1e+03
mean of normalized counts

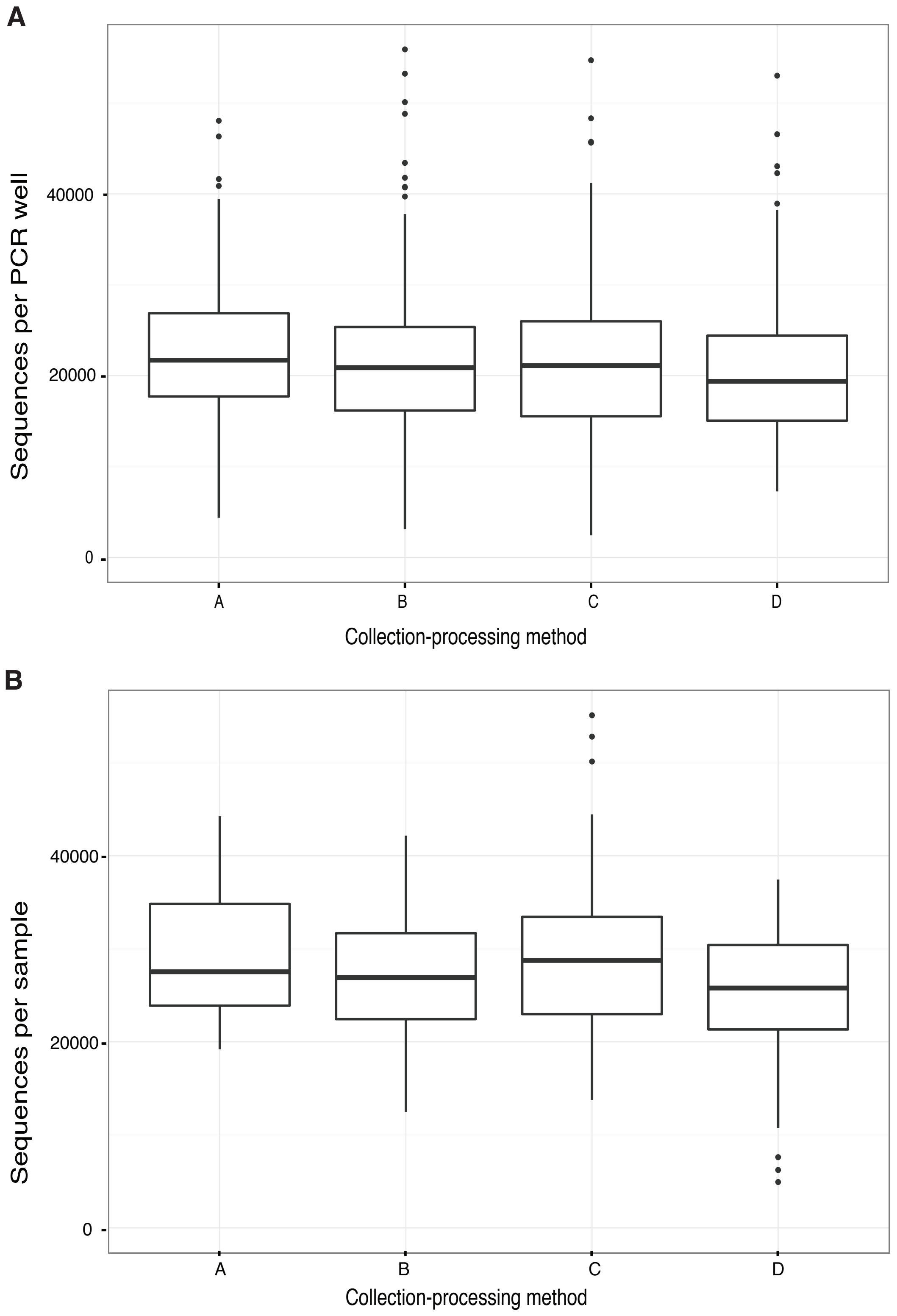
A
Sequences per PCR well
40000
20000
0
A
B
C
D
Collection-processing method
B
Sequences per sample
40000
20000
0
A
B
C
D
Collection-processing method

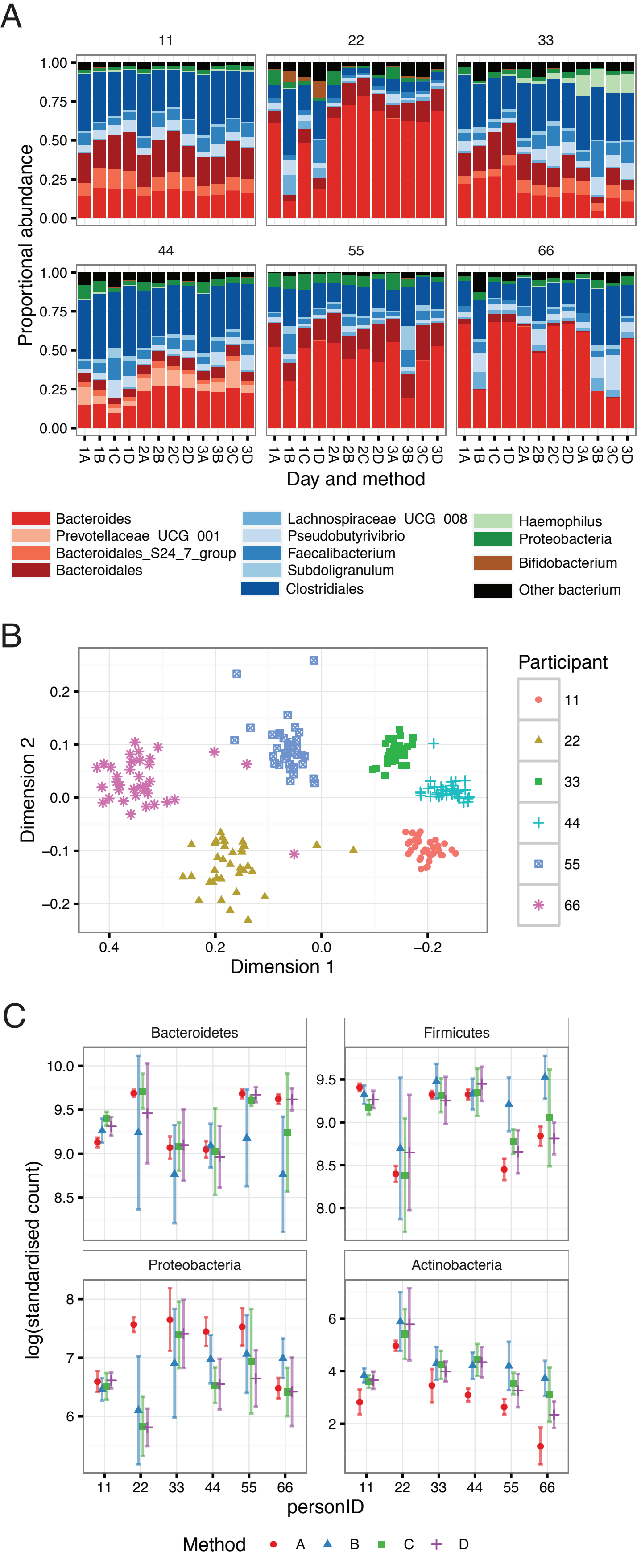
A
11
22
33
44
55
66
Proportional abundance
1.00
0.75
0.50
0.25
0.00
1A 1B 1C 1D 2A 2B 2C 2D 3A 3B 3C 3D
Day and method
Bacteroides
Prevotellaceae_UCG_001
Bacteroidales_S24_7_group
Bacteroidales
Lachnospiraceae_UCG_008
Pseudobutyrivibrio
Faecalibacterium
Subdoligranulum
Clostridiales
Haemophilus
Proteobacteria
Bifidobacterium
Other bacterium
B
Dimension 2
0.2
0.1
0.0
−0.1
−0.2
0.4
0.2
0.0
−0.2
Dimension 1
Participant
11
22
33
44
55
66
C
Bacteroidetes
Firmicutes
Proteobacteria
Actinobacteria
log(standardised count)
10.0
9.5
9.0
8.5
8.0
8
7
6
4
2
11 22 33 44 55 66
personID
Method
A
B
C
D

Table S1 (A) Differences by collection-processing method in phyla and OTU counts (WEHI and BCM data sets). No collection-processing method had significantly different phyla in the WEHI data set

Method comparison	# OTUs different (WEHI)	# phyla different (BCM)	# OTUs different (BCM)
**A-D**	1	1	26
**B-D**	0	0	0
**C-D**	0	0	0
**B-A**	0	2	51
**C-A**	5	1	25
**C-B**	0	0	0

Wald test for difference of size of log2 (fold change) > 1 with adjusted p-value < 0.05. Total number of OTUs with minimum count of 20: WEHI 12,764; BCM 3,675.

Table S1 (B) Phyla different between collection-processing methods in BCM data set

Phylum	baseMean	Comparison	$\log_2$ (fold change)	adjusted p
Actinobacteria	123.1	Method A vs B	-1.7	6×10^{-4}
Lentisphaerae	4.2	Method A vs B	2.2	9×10^{-4}
		Method A vs C	2.0	0.011
		Method A vs D	2.2	0.003

Table S1 (C) OTUs different between collection-processing methods in WEHI data set

OTU-ID	Taxonomic Family	BaseMean	Comparison	$\log_2$ (fold change)	adjusted p
185034	Lachnospiraceae	2.0	Method A vs C	2.1	2.5×10^{-6}
			Method A vs D	2.1	2.5×10^{-6}
573110	Lachnospiraceae	8.3	Method A vs C	1.8	1.1×10^{-5}
351659	Lachnospiraceae	22.2	Method A vs C	1.6	1.2×10^{-5}
180713	Ruminococcaceae	4.0	Method A vs C	-2.1	5.3×10^{-7}
367213	Ruminococcaceae	61.5	Method A vs C	-1.9	1.3×10^{-7}

Table S2: Effect of Method on Beta Diversity

Table S2(A) WEHI sequencing

Method comparison	mean difference	95% confidence lower bound	95% confidence upper bound	family-wise adjusted p-value
**B-A**	0.00	-0.02	0.03	0.99
**C-A**	-0.01	-0.04	0.01	0.52
**D-A**	-0.02	-0.05	0.01	0.22
**C-B**	-0.02	-0.05	0.01	0.33
**D-B**	-0.02	-0.05	0.00	0.11
**D-C**	-0.01	-0.03	0.02	0.94

Table S2(B) BCM sequencing

Method comparison	mean difference	95% confidence lower bound	95% confidence upper bound	family-wise adjusted p-value
**B-A**	0.15	0.12	0.19	0.000
**C-A**	0.10	0.07	0.14	0.000
**D-A**	0.14	0.10	0.17	0.000
**C-B**	-0.05	-0.09	-0.02	0.000
**D-B**	-0.02	-0.05	0.02	0.592
**D-C**	0.04	0.00	0.07	0.036

Metric is Bray-Curtis distance between samples from same participant with same collection-processing method. Analysis of variance based on a linear model of pairwise distance as a function of Method and Participant
